# MoSe_2_-WS_2_ Nanostructure for an Efficient Hydrogen Generation under White Light LED Irradiation

**DOI:** 10.3390/nano12071160

**Published:** 2022-03-31

**Authors:** Tatiparti Padma, Dheeraj Kumar Gara, Amara Nadha Reddy, Surya Veerendra Prabhakar Vattikuti, Christian M. Julien

**Affiliations:** 1 Department of Electronics & Communications Engineering, Gokaraju Rangaraju Institute of Engineering and Technology, Kukatpally, Hyderabad 500090, Telangana, India; profpadmat@gmail.com; 2 Malla Reddy College of Engineering and Technology, Doolapally, Hyderabad 500100, Telangana, India; dheeru498@gmail.com (D.K.G.); amarnadha@gmail.com (A.N.R.); 3 School of Mechanical Engineering, Yeungnam University, Gyeongsan 38541, Korea; vsvprabu@gmail.com; 4 Institut de Minéralogie, de Physique des Matériaux et de Cosmochimie (IMPMC), Sorbonne Université, CNRS-UMR 7590, 4 Place Jussieu, 75252 Paris, France

**Keywords:** hydrogen production, layered materials, photocatalysts, WS_2_, MoSe_2_

## Abstract

In this work, MoSe_2_-WS_2_ nanocomposites consisting of WS_2_ nanoparticles covered with few MoSe_2_ nanosheets were successfully developed via an easy hydrothermal synthesis method. Their nanostructure and photocatalytic hydrogen evolution (PHE) performance are investigated by a series of characterization techniques. The PHE rate of MoSe_2_-WS_2_ is evaluated under the white light LED irradiation. Under LED illumination, the highest PHE of MoSe_2_-WS_2_ nanocomposite is 1600.2 µmol g^−1^ h^−1^. When compared with pristine WS_2_, the MoSe_2_-WS_2_ nanostructures demonstrated improved PHE rate, which is 10-fold higher than that of the pristine one. This work suggests that MoSe_2_-WS_2_ could be a promising photocatalyst candidate and might stimulate the further studies of other layered materials for energy conversion and storage.

## 1. Introduction

Presently, energy shortage and environmental pollution are the major problems [1,2,3]. Intuitively, the current photocatalytic hydrogen (H_2_) generation from water through semiconductor nanostructures is an ideal approach, and this technique is considered as a potential, economical way to overcome the issue of energy shortage [4,5,6,7]. Over the years, there has been substantial experimental data calibrated on transition-metal (TM) oxide-based photocatalysts for energy-storage applications [6,7,8] with minimal environmental pollution. Though these materials display excellent photocatalytic activity just beneath the UV illumination, they exhibit poor activity in visible light due to their wide bandgap. In order to stimulate photocatalytic activity in visible light, recent attempts on anion-doped TM oxides-, sulfides-, and selenides-based nanostructures have been reported [9,10]. However, these TMs are unstable, making them inert towards commercial applications. Henceforth, there is a challenge for the researchers to identify and develop potential, stable and economically novel photocatalysts for H_2_ evolution.

In lieu of this, the two-dimensional (2D) MoSe_2_ semiconductor is a compelling potential catalyst for next-generation hydrogen evolution due to its narrow bandgap (1.2 eV), specific surface area and more metallic nature, which prompts higher electrical conductivity that is more favorable to hydrogen evolution reactions. However, there are few reports on the preparation of MoSe_2_-based composites through diverse approaches, which display an enhanced photocatalytic activity (PCA) when compared with the bare MoSe_2_ [11]. In spite of this enhanced photocatalyst, there is provision to promote further the PCA in the water-splitting process by resolving the photo corrosion and faster charge recombination problems. In this context, an inclination towards the development of a nanostructure heterojunction, which can improve the visible light absorption, further altering the surface defects followed by reduction in the surface recombination. This may enhance the capability of water splitting both quantitatively and qualitatively. Recently, 2D-layered WS_2_ has enticed significant attention in the field of energy and photocatalysis applications because of its promising characteristics towards photocatalytic performance [11,12]. There is a decent work available in the scientific literature on WS_2_-based composites, which displayed significant enhancement in the PCA compared to pristine WS_2_ [12,13,14]. Various methods have been reported in the literature to process these composites [15,16]. However, in addition to this, the interest of an advanced study combining two transition-metal dichalcogenides (TMDs) such as MoSe_2_ and WS_2_ possessing alike hexagonal structure, enables to simulation of the heterojunction formation.

The MoX_2_/WX_2_ (X = S, Se) heterostructures, which exhibit a type-II band configuration, are considered to be efficient systems for the production of optoelectronic and photovoltaic devices, in which the free electrons and holes are spontaneously isolated [17]. The lattice constants of MoX_2_ and WX_2_ being very close to each other indicate that MoX_2_-WX_2_ heterostructures can have minimum structural defects. According to the theoretical calculations, the conduction band minimum (CBM) of the WS_2_ is only slightly lower than that of the MoSe_2_ [18]. In 2018, Jin et al. reviewed experimental and theoretical efforts to elucidate electron dynamics in TMDC heterostructures [19]. The dominant interlayer electron transport relaxation pathway in WS_2_/MoSe_2_ heterostructures proving the strong interlayer dipole−dipole interaction was identified by Kozawa et al. [20]. Photoluminescence excitation spectroscopy evidenced the fast interlayer energy transfer across the van der Waals interface of the MoSe_2_/WS_2_ heterostructures. Meng et al. investigated the ultrafast carrier transfer, which can efficiently separate electrons and holes in the intralayer excitons in a MoSe_2_/WS_2_ heterostructure [21]. Based on first-principles ab initio calculations, Amin et al. investigated the band structure of WS_2_/MoSe_2_ and showed the indirect electron transition semiconducting behavior [17]. The distinctive interactions between stacked layer are essential for solar cells because the confinement of electrons by MoSe_2_ and holes by WS_2_, leading to a spontaneous charge separation when excitons scatter to the WS_2_/MoSe_2_ junction [22]. Ceballos et al. evidenced highly efficient and anomalous charge transfer in the van der Waals MoSe_2_/WS_2_/MoS_2_ trilayer semiconductors [23]. Wu et al. fabricated a WS_2_/MoSe_2_ hybrid semiconductor catalyst (WS_2_ mass fraction of 20%) with a p-n heterojunction, which is composed of spherical WS_2_ particles (2 µm diameter) mixed with flower-like granularMoSe_2_ (50 nm in size). This product exhibits good photocatalytic performance with a photocurrent density of 35 μA cm^−2^ at −0.6 V vs. SCE [24].

Moreover, experimental and theoretical investigations have shown that the unsaturated X-edges of TMDs are favorable to hydrogen evolution electrocatalytic activity [25]. Band offsets and heterostructures of monolayer and few-layer TMDs were calculated using the vacuum level as reference, and a simple model was proposed to explain the observed chemical trends [26]. This concept has been applied to the MoS_2_/WS_2_ and MoS_2_/WSe_2_ heterostructures, which exhibited robust electrocatalytic properties [27]. Recently, Vikraman et al. have constructed a MoSe_2_/WS_2_ heterojunction model by a chemical/physical process and have intricately examined its hydrogen evolution reaction performances [28]. The MoSe_2_/WS_2_ heterostructure displayed excellent electrocatalytic hydrogen evolution behavior with a 75 mV overpotential to drive a 10 mA·cm^−2^ current density, a 60 mV·dec^−1^. Such a type of structure is developed as an active electrode for hydrogen evolution to replace the nonabundant Pt.

To the best of our knowledge, both photocatalytic hydrogen evolution (PHE) and electrocatalytic hydrogen evolution (EHE) performance of the van der Waals two-layer MoSe_2_-WS_2_ heterostructure has not been studied yet. Only a mixture of WS_2_ and MoSe_2_ particles was considered. The main goal of the present work was to synthesize a MoSe_2_-WS_2_ nanocomposite through hydrothermal process and further elucidate its photocatalytic and electrocatalytic properties for hydrogen evolution. As anticipated, the MoSe_2_-WS_2_ nanostructure displayed better PHE rate that of pristine WS_2_ sample. It is demonstrated that the PHE of MoSe_2_-WS_2_ is 10-fold higher than that of WS_2_.

## 2. Materials and Methods

The raw materials sodium tungstate dehydrates, polyethylene glycol, and thioacetamide were purchased from Sigma-Aldrich (St. Louis, MO, USA). Ammonium molybdate tetrahydrate was from Junsei Chemical Co. (Tokyo, Japan). Polyvinylpyrrolidone (PVP), Selenium powder and anhydrous oxalic acid were provided from Daejung (Daejeon, Korea) and used as received without further treatment.

### 2.1. Preparation of WS_2_

The synthesis procedure for WS_2_ followed that of Yuan et al. [29] with modification. Typically, 1.1 g of sodium tungstate dehydrate, 2.3 g of thioacetamide, and 5.5 mL of polyethylene glycol were dissolved in 25 mL of deionized water followed by stirring to obtain a homogeneous solution. Then, the pH of the solution was adjusted to 2 by adding the oxalic acid and transferred to sealed autoclave-reactor solution that was maintained at 210 °C for 48 h. After cooling, the precipitates were collected by centrifuge at 9000 rpm, washed with water and ethanol for several times, and dried in a vacuum oven for 100 °C for 48 h and then annealed at 600 °C for 1 h under Ar to obtain the final product.

### 2.2. Preparation of MoSe_2_-WS_2_

Typically, 0.025 mol of NH_4_Mo_7_O_24_∙4H_2_O and 0.12 g of PVP were dissolved in 12 mL of ammonium hydroxide solution under constant stirring. On the other hand, 0.05 mol of Se powder was dispersed in hydrazine hydrate under vigorous stirring for 30 min. Then, this solution was added dropwise to the above resultant solution at room temperature, which has a nominal Mo/Se molar ratio of 1:2. After that, 25 mg of WS_2_ was added into resultant solution stirred for about 1 h. The resulting homogenous solution was irradiated by hydrothermal reaction at 220 °C for 48 h. The as-obtained precipitate (MoSe_2_-WS_2_) was collected by centrifugation at 9000 rpm, washed with a mixture of deionized water and ethanol, and dried in a vacuum oven at 130 °C for 48 h. To make a comparison, pure MoSe_2_ was also synthesized from the similar procedure without WS_2_.

### 2.3. Instruments

X-ray diffraction (XRD) patterns were analyzed on Shimadzu 6100 X-ray diffractometer (Shimadzu Corp., Tokyo, Japan) equipped with a CuK_α_ X-ray source (λ = 1.5406 Å). The morphology analysis was performed by transmission electron microscopy (TEM), high-resolution TEM (HRTEM) and high-angle annular dark field-scanning transmission electron microscopy (HAADF-STEM) using a FEI Tecnai G2 F20 (FEI Company, Hillsboro, OR, USA). Initially, the synthesized MoSe_2_-WS_2_ nanocomposites were dispersed in ethanol and sonicated for 10 min, then the dispersed MoSe_2_-WS_2_ was dropped on a TEM grid (200 mesh Cu grid) and dried at 90 °C overnight.

The X-ray photoelectron spectra (XPS) were recorded using a Thermo Scientific k-α surface analyzer (ThermoFisher Scientific, Alachua, FL, USA), equipped with a AlK_α_ X-ray source (hν = 1486 eV). The ultraviolet–visible (UV-Vis) spectroscopy was carried out on a Cary 5000 (ThermoFisher Scientific, Alachua, FL, USA) in the 200–800 nm wavelength range using a transmittance and reflectance equipment. The infrared spectra were recorded in the spectral range of 400–4000 cm^−1^ using an Avatar 370 Fourier transform infrared spectrometer (FTIR, Thermo Nicolet, Alachua, FL, USA). The Brunauer-Emmett-Teller (BET) method was used to determine the specific surface area and pore size distribution measured by an ASAP 2420 surface area analyzer degassed for 1 h at 150 °C (Micromeritics, Norcross, GA, USA). High-resolution transmission electron microscope (HRTEM Titan G2 Chemi STEM Cs probe, FEI Company, Hillsboro, OR, USA) with EDS windowless (Super-X model) and physisorption analyzer (BET-Micromeritics, 3FLEX, Norcross, GA, USA) were used for both morphology and surface area studies.

### 2.4. Photocatalytic Hydrogen Tests

The photoreaction was conducted in a quartz top-irradiation reactor tightened with silicone rubber “Septa”. Then, 5 mg of photocatalysts and 5 mL of Na_2_S/Na_2_SO_3_ were dispersed into 45 mL of water by sonication for 40 min. The reactor was evacuated for 45 min and N_2_-bubbled for 30 min prior to irradiation. A light-emitting diode (LED, white light irradiation) was facilitated by 100 W lamp without an optical filter. The induced gases were measured using a gas chromatograph (YL-6500 with TCD detector) equipped with a 5-Å molecular sieve column under He carrier gas.

### 2.5. Electrochemical Measurements

Electrochemical characterizations were performed with a Bio-Logic (SP-200) workstation with a standard three-electrode cell using Ag/AgCl (3.5 mol L^−1^ KCl solution), a platinum coil and fluorine-doped tin oxide (FTO) glass as reference, counter and working electrodes, respectively. The working electrode was prepared as follows: 5 mg of catalysts and 80 µL of 5 wt.% Nafion solution were dispersed in 1 mL of a solution of deionized water and ethanol, i.e., 4:1 in volume ratio, and then stirred for 45 min. A total of 5 µL of the resultant solution was drop-casted on the FTO glass. The working electrode was dried at 75 °C for 1 h. Photocurrent measurements and electronchemical impedance spectroscopy analyses were carried out in 0.5 mol L^−1^ Na_2_SO_4_ solution with purging nitrogen gas.

## 3. Results and Discussion

### 3.1. XRD Analysis

**Figure 1:**
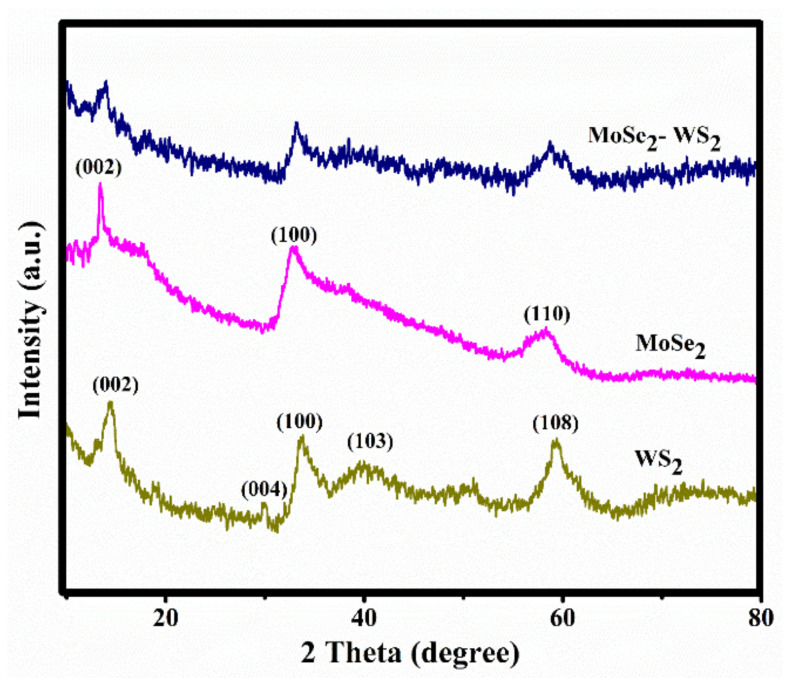
XRD patterns of pristine WS_2_ and MoSe_2_, and nanostructured MoSe_2_-WS_2_ composite. Spectra were recorded using a CuK_α_ X-ray source (λ = 1.5406 Å).

The phase purity and crystal structure of the as-prepared nanostructures were analyzed through bulk X-ray diffraction. Figure 1 displays the XRD patterns of WS_2_, MoSe_2_ pristine materials and that of the MoSe_2_-WS_2_ nanocomposite in the 2θ range 10–80°. The diffraction peaks at 13.7°, 28.21°, 34.11°, 39.49°, and 58.89° corresponding to the (002), (004), (100), (103), and (008) crystal planes, respectively, match well with the hexagonal structure of WS_2_ (JCPDS Card # 08-0237) [29,30,31]. 

The as-synthesized MoSe_2_ similarly displays a hexagonal structure and XRD patterns are in good agreement with literature data (JCPDS Card # 29-0914) [32]. No other impurity peaks were detected. The peak positions of the as-prepared MoSe_2_-WS_2_ sample are identical to those of WS_2_ showing that layered MoSe_2_ does not substitute into the WS_2_ lattice. Although, an obvious decrease in the peak intensity is noticed, while no peaks related to MoSe_2_ are recognized due to its good dispersion and its low loading content.

### 3.2. Morphology and Elemental Analysis

**Figure 2:**
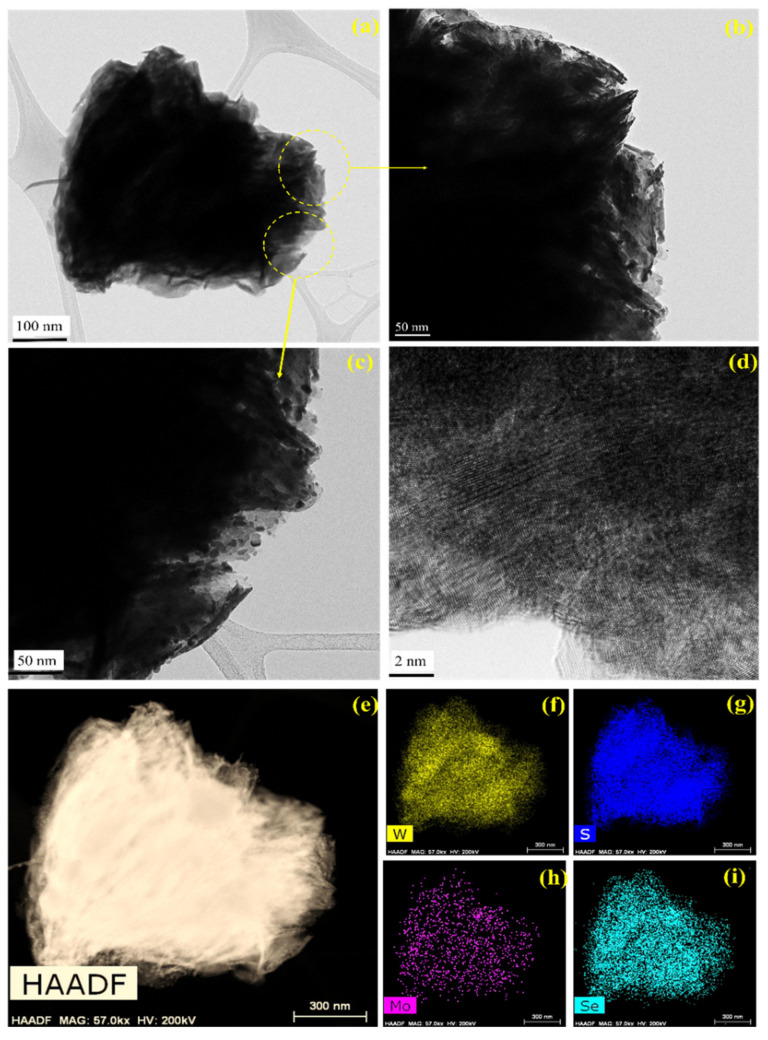
(**a**–**c**) TEM images of the MoSe_2_-WS_2_ nanocomposite, (**d**) HRTEM image, (**e**) HAADF-STEM pattern, and (**f**–**i**) corresponding elemental mapping images of W, S, Mo and Se.

HRTEM was employed to probe the morphology of the as-synthesized MoSe_2_-WS_2_ nanocomposite (Figure 2a–d). As depicted, the typical sheet-like structure of MoSe_2_ can be distinctly noticed. Some MoSe_2_ slabs with the characteristic layered structure are remarkable on the surface of WS_2_ particles (see Figure 2b–d). In addition, the HAADF-STEM elemental mapping images shown in Figure 2e–i clearly depict that W (yellow), Mo (purple), S (dark blue), and Se (light blue) are uniformly distributed on the surface of nanostructures. These findings indicate that the MoSe_2_-WS_2_ nanocomposite heterostructures were effectively prepared.

### 3.3. XPS Analysis

**Figure 3:**
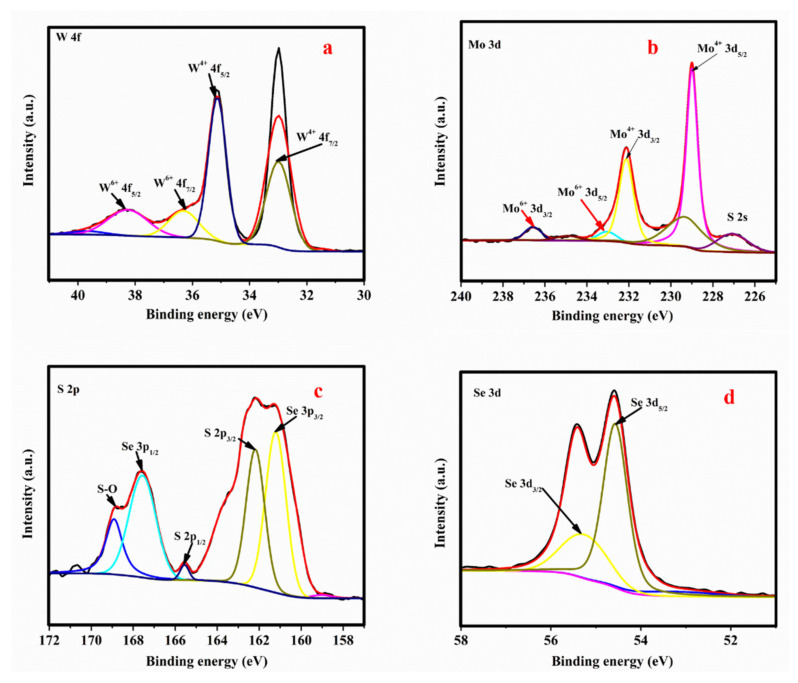
XPS elemental profiles of (**a**) W, (**b**) Mo, (**c**) S, and (**d**) Se elements of the MoSe_2_-WS_2_ nanostructure.

Figure 3 presents the XPS magnified scans of (a) W, (b) Mo, (c) S and (d) Se of the MoSe_2_-WS_2_ sample and confirms the existence of MoSe_2_ and WS_2_. Figure 3a depicts the W 4f peaks at binding energies of 32.1, 34.1, 36.2, and 28.3 eV corresponding to the W^4+^ 4f_7/2_, W^4+^ 4f_5/2_, W^6+^ 4f_7/2_, and W^6+^ 4f_5/2_ core level, respectively, which indicates the existence of W^4+^, W^6+^ species in the synthesized MoSe_2_-WS_2_ [31]. The higher binding energy of 36.2 eV signifies the existence of the +6 oxidation state of W element, with a very low peak intensity specifying that only a small portion of W^4+^ cations is oxidized when the sample is in contact with the air [32]. Figure 3b shows the Mo 3d contribution with peaks located at 228.0 and 232.6 eV, belonging to the Mo^4+^ 3d_5/2_ and Mo^4+^ 3d_3/2_ core level of MoSe_2_. The valence states of the Mo element corresponding to the double peaks at 231.2 and 235.1 eV are assigned to Mo^6+^ 3d_5/2_ and Mo^6+^ 3d_3/2_ core levels specifying the oxidized states of Mo due to air contact [16,17]. The S 2p peak can be deconvoluted into two peaks at 163.6 and 161.8 eV (Figure 3c) corresponding to the S 2p_1/2_ and S 2p_3/2_ core levels with doublet positioned at 166.98 and 160.97 eV, which can be attributed to the Se 3p_1/2_ and Se 3p_1/2_ core levels [33]. Finally, Figure 3d displays the binding energies of Se 3d_5/2_ at 54.3 eV and Se 3d_3/2_ at 55.2 eV, corresponding to the divalent (−2) state of Se. All these values match well with those reported in previous investigations and indicate the anticipated chemical states of Mo^4+^, W^4+^, Se^2-^, and S^2^^−^ in the MoSe_2_-WS_2_ nanostructure [31].

### 3.4. FTIR Analysis

**Figure 4:**
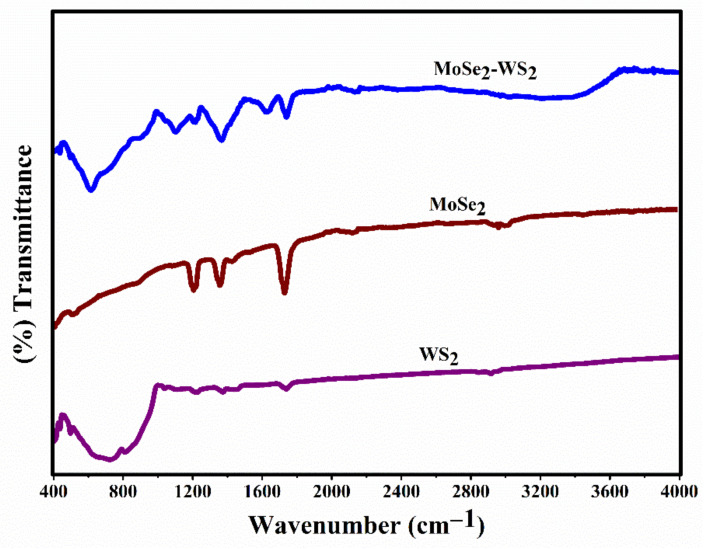
FTIR spectra of WS_2_, MoSe_2_, and MoSe_2_-WS_2_ catalysts.

Figure 4 shows the FTIR spectra of WS_2_, MoSe_2_ and MoSe_2_-WS_2_ samples recorded in the range 400–4000 cm^−1^. In the FTIR spectrum of WS_2_, the bands pointed at 743, 1739 and 2943 cm^−1^ are attributed to the W-S bending and stretching vibrations, and hydroxyl groups related to O-H bonds, respectively [33,34]. In the MoSe_2_ spectrum, the bands located at 523, 1218, 1327, 1744 and 3013 cm^−1^ could be ascribed to the stretching vibration of Mo-Se and hydroxyl groups of MoSe_2_ [35]. For the MoSe_2_-WS_2_, the band intensities corresponding to the hydroxyl group decrease significantly, and the infrared band of the Se-W-S bonds shifts from 743 to 622 cm^−1^, which suggests the interface interaction between the MoSe_2_ and WS_2_.

### 3.5. Surface Area Analysis

**Figure 5:**
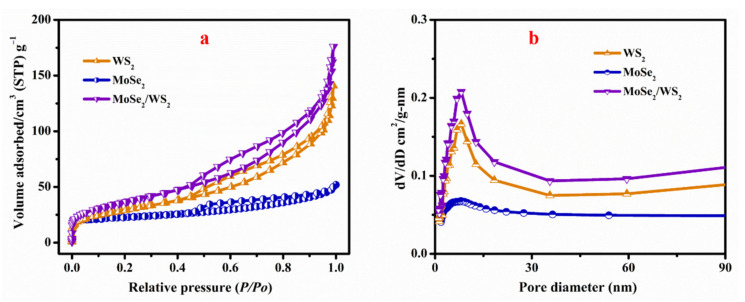
(**a**) BET profiles and (**b**) pore distribution of pristine WS_2_, MoSe_2_, and MoSe_2_-WS_2_ samples.

Figure 5a shows the N_2_ adsorption–desorption isotherms of pristine WS_2_ and MoSe_2_-WS_2_ samples. Both samples show the standard type-IV isotherms with obvious hysteresis loops, endorsing the presence of hierarchical mesoporosity [36]. On the other hand, the sharp uptakes at high pressure 0.55–1.0 *P*/*P*o of WS_2_ and MoSe_2_-WS_2_ indicate the presence of mesopores in both samples, which is in good agreement with the results of pore size distribution curves as shown in Figure 5b. Data are listed in Table 1. The MoSe_2_-WS_2_ nanocomposite has a high specific surface area of 132.79 m^2^ g^−1^, which is slightly higher than pristine WS_2_ (106.23 m^2^ g^−1^). On the other hand, both samples exhibit almost the same pore size: 10.6(5) nm for MoSe_2_-WS_2_ and 10.7(2) nm for WS_2_.

### 3.6. Optical Studies

**Figure 6:**
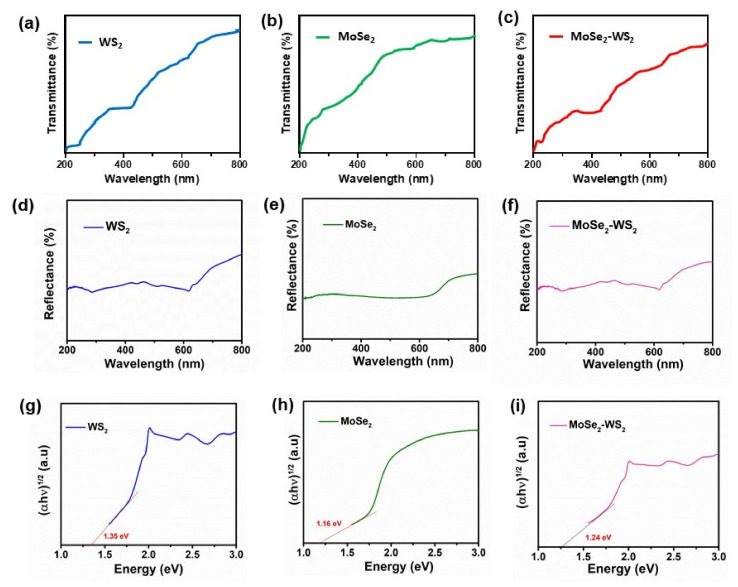
Determination of the bandgap of WS_2_, MoSe_2_ and MoSe_2_-WS_2_ catalysts. (**a**–**c**) UV-Vis transmittance spectra, (**d**–**f**) UV-Vis reflectance spectra and (**g**–**i**) Tauc’s plots.

To evaluate the electronic and optoelectronic properties of the MoSe_2_-WS_2_ nanocomposite, the optical bandgap has been determined using the UV-Vis spectroscopy in the vicinity of the fundamental transition, i.e., wavelength range of 200–800 nm, and compared with that of pristine WS_2_ and MoSe_2_ samples. The UV–Vis transmittance and reflectance spectra of samples are depicted in Figure 6a–f. Analyses of material bandgaps are presented in Figure 6g–i.

The optical absorption coefficient (α) is calculated taking into consideration the transmittance (*T*%) and reflectance (*R*%) of the sample using the standard equation [37]:

(1) $$\alpha =\frac{1}{d}\;ln\left[\frac{{\left(1-R\right)}^{2}}{T}\right],\;$$

where *d* is the film thickness. The bandgap *E*_g_ of samples is calculated by the Tauc’s formula [38]:

*αhν* = *C* (*E*_g_ − *hν*)*^n^*, (2)

where *h* is the Plank constant, ν is the photon energy, *E*_g_ is the average bandgap of the material, *C* is a constant depending on several intrinsic properties of the material, i.e., the effective mass of the electron and hole and the material refractive index, and *n* is the transition-type dependent. It is equal to 1/2, 3/2, 2 and 3 for the direct-allowed, direct-forbidden, indirect-allowed and indirect-forbidden transitions, respectively [39]. The average bandgap was calculated from the intercept of the linear part of the (*αhν*)^2^ vs. *hν* plot on *x*-axis for all samples as shown in Figure 6g–i.

The evaluated optical bandgap values (±0.02 eV) are 1.35, 1.16 and 1.24 eV for WS_2_, MoSe_2_ and MoSe_2_-WS_2_, respectively. The knowledge of the bandgap is also useful for the phase identification of the samples. It has been demonstrated that 2D TMDs possess sizable bandgaps around 1–2 eV [40]. According to Wang et al. [41], the bandgap of TMDs has the following electronic properties: (i) the bulk has an indirect bandgap of 1.3 eV for WS_2_ and 1.1 eV for MoSe_2_ and (ii) the monolayer has a direct bandgap of 2.1 eV for WS_2_ and 1.5 eV for MoSe_2_. Thus, the bandgap of the MoSe_2_-WS_2_ nanocomposite, in which WS_2_ is the core of the sample (bulk) and MoSe_2_ is formed by few nanosheets covering the bulk is in good agreement with previous reports [36]. Both MoSe_2_ and WS_2_ layered compounds are expected to undergo a similar indirect-to-direct bandgap evolution with decreasing layer numbers [42]. Indeed, the optical properties of the samples critically depend on the physical properties, and these variations in the energy gap here are consistent with those of the crystallite size.

### 3.7. Photocatalytic Performance

**Figure 7:**
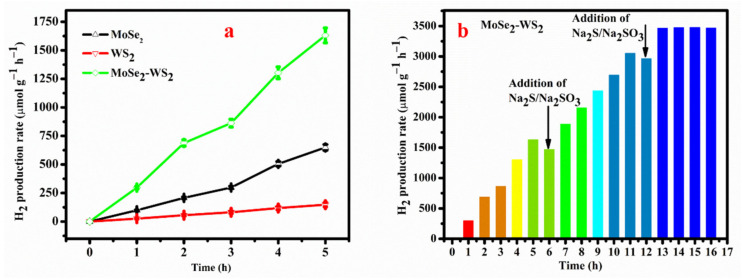
(**a**) H_2_ generation rate of MoSe_2_, WS_2_, and MoSe_2_-WS_2_ catalysts and (**b**) continuous cycling stability of MoSe_2_-WS_2_ catalyst under visible light irradiation.

The photocatalytic performances of the as-synthesized products were estimated through water splitting for H_2_ evolution. Figure 7a displays the comparison of hydrogen evolution performance of MoSe_2_, WS_2_ and MoSe_2_-WS_2_ nanostructures for 5 h under LED irradiation. The achieved H_2_ production is 600.1, 150.2 and 1600.2 µmol g^−1^ h^−1^ for MoSe_2_, WS_2_, and MoSe_2_-WS_2_, respectively. The obtained H_2_ generation for MoSe_2_-WS_2_ is almost 3 and 10 times higher than that of bare MoSe_2_ and WS_2_, respectively. It is vital to consider that all the photocatalytic experimental results in this work were employed in an identical condition. The enhanced photocatalytic activity of MoSe_2_-WS_2_ nanostructure has the following characteristics: (i) it is due to the formation of heterostructures, which promote the separation of the photocarriers a well as their recombination, (ii) the coupling of MoSe_2_ and WS_2_ with diverse energy levels could engender the enhancement of the separation and transfer of photoinduced charges, which leads the photoinduced carriers to involve in the photo-redox reactions, and (iii) the MoSe_2_ acts as a cocatalyst for generation of H_2_. On the other hand, after a few hours, the volume of the reactor became insufficient to accommodate a large amount of H_2_, which increased solubility of H_2_ in the solution, suppressing the production of H_2_ in solution, reducing the evolution of H_2_ and total scavenger consumption, reducing the ability of H_2_ evolution [43]. Thus, it needs to provide additional number of scavengers in the continuous time-on-stream activity of the catalysts. Figure 7b illustrates the continuous stability of MoSe_2_-WS_2_ nanostructure over 16 h. After 6 h of continuing H_2_ production, it was observed that changing the scavenger and adding scavengers would increase H_2_ production, then reduced H_2_ production at 11 h and adding the scavenger again at 12 h, the increase in H_2_ activity was maintained steadily until 16 h on MoSe_2_-WS_2_ nanostructure.

These results suggest that the H_2_ evolution of the MoSe_2_-WS_2_ nanostructure harbors good stability up to three cycles. Therefore, MoSe_2_-WS_2_ nanostructures are promising and potential candidates for practical photocatalytic H_2_ evolution. Note that the as-prepared MoSe_2_-WS_2_ nanocomposite shows better H_2_ production yield (10 times greater than that bare WS_2_) than that of the WS_2_-MoS_2_ heterostructure (6.5 times greater than that bare WS_2_) [43].

### 3.8. Electrochemical Performance

**Figure 8:**
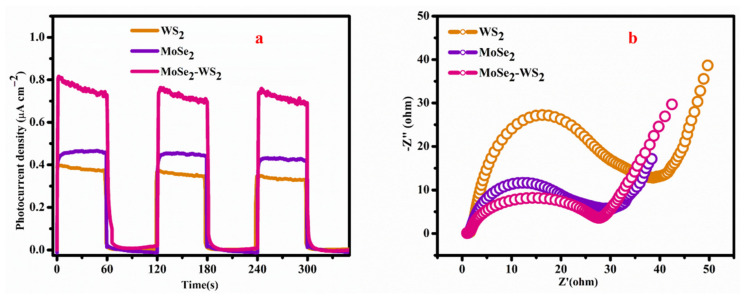
(**a**) Photocurrent response and (**b**) EIS spectra of WS_2_, MoSe_2_ and MoSe_2_-WS_2_ nanostructure.

To probe the enriched mechanism of the photocatalytic H_2_ production, the excitation and transfer of photogenerated charge carriers of the as-synthesized products were studied. The photocurrent-time (PI) as well as the electrochemical impedance spectroscopy (EIS) were employed. The acquired PI profiles are displayed in Figure 8a, which portrays the periodic on-off photocurrent response of all the prepared products under visible light illumination. 

Identically, the photocurrent response of MoSe_2_-WS_2_ is higher than that of bare WS_2_, which is consistent with the photocatalytic activity. Measurements carried out in 0.5 mol L^−1^ Na_2_SO_4_ solution show a photocurrent density of 0.75 µA cm^−2^ for the MoSe_2_-WS_2_ heterostructure against 0.38 µA cm^−2^ for bulk WS_2_. This result demonstrated that the MoSe_2_-WS_2_ nanocomposite has brawny ability in transferring and generating the photo-excited charge carrier under the visible light illumination. On the other hand, recombination process and charge transfer of photo-induced electrons as well as holes can be displayed via EIS (Figure 8b). Compared with that of bare MoSe_2_ and WS_2_ materials, the Nyquist plot evidences clearly a depressed semicircle for MoSe_2_-WS_2_, which designates a fast charge-carrier transfer rate in the MoSe_2_-WS_2_. Hence, it stipulates that the effective transfer of photo-induced electrons among MoSe_2_ and WS_2_ enables the electron–hole separation.

### 3.9. Proposed Photocatalytic H_2_ Mechanism

**Figure 9:**
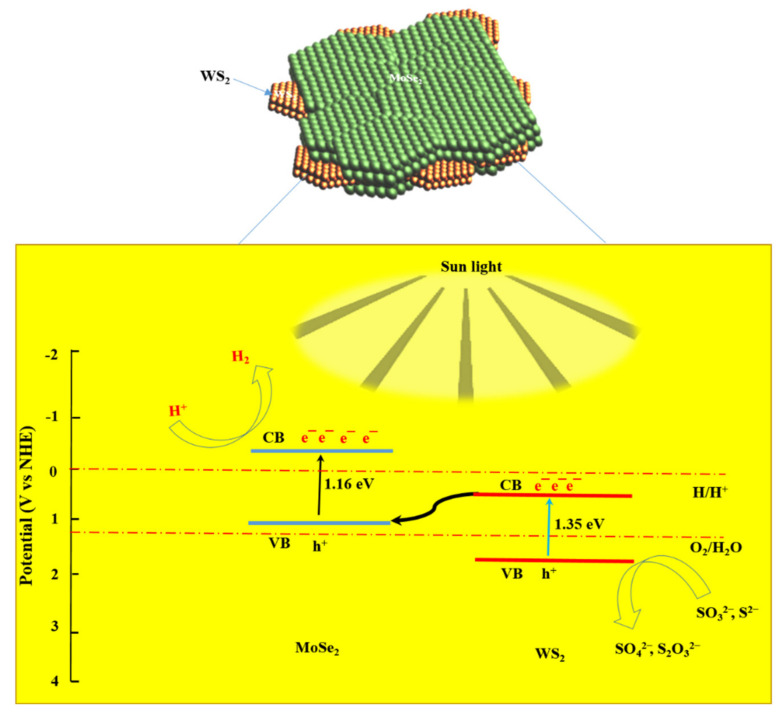
Scheme of the photocatalytic mechanism of MoSe_2_-WS_2_ nanostructure under light irradiation.

In pursuant with the results discussed above, a mechanism for water reduction by the use of MoSe_2_-WS_2_ nanostructure-based catalyst is proposed as illustrated by the scheme in Figure 9. The electron–hole pairs are usually generated on MoSe_2_-WS_2_ under the visible light illumination. In the development of visible-light-driven devices, the nanostructured MoSe_2_-WS_2_ composite is a so-called Z-scheme photocatalyst [44,45,46,47]. In this system, electrons are excited from the valence band (VB) to the conduction band (CB) of WS_2_ upon visible light illumination, then transferred to VB of MoSe_2_ and finally reach to CB of MoSe_2_ during generation of H_2_. The photocatalytic H_2_ mechanism has been described as follows: in the H_2_ evolution setup, the photoreduction of proton by CB electrons as:

2H^+^ + 2e^−^ → H_2_, (3)

and the oxidation of an electron donor (D) by VB holes yields an electron acceptor (A) as:

D + nh^+^ → A. (4)

Thus, the water-splitting reaction occurs when a cycle of D and A redox pairs is achieved. Meanwhile, the photogenerated holes on the VB of WS_2_ can reduce the scavengers, which considerably decrease the recombination process of electron–hole pairs and lead to improved stability of the MoSe_2_-WS_2_ nanostructure and the enhancement of hydrogen production rate. Consequently, the photocatalytic H_2_ evolution activity is promoted for WS_2_ modified with few MoSe_2_ monolayers, which promotes a significant increase of photogenerated charge–hole separation efficiency [48].

## 4. Conclusions

In summary, we have investigated the structural, vibrational and electronic characteristics of the van der Waals interrelated MoSe_2_-WS_2_ nanostructure used as photocatalysts for H_2_ production. We found that:

(1) A cost-effective and simple chemical methodology was handled to fabricate the MoSe_2_-WS_2_ nanostructure using an easy one-step hydrothermal process without high-temperature annealing;
(2) The MoSe_2_-WS_2_ nanocomposite has a high specific surface area of 132.79 m^2^ g^−1^ and a pore size of 10.6 nm, which are values favorable for an efficient photocatalytic activity. For the MoSe_2_-WS_2_ heterostructure, in which WS_2_ is the core of the sample (bulk) and MoSe_2_ is formed by a few nanosheets covering the bulk, the evaluated optical bandgap is 1.24 eV;
(3) The use of MoSe_2_ and WS_2_ sheets with similar lattice parameters allows the fabrication of heterostructure without matching restriction;
(4) The coupling of MoSe_2_ with WS_2_ led to a considerably enhanced surface area and higher photoinduced charge separation. It results a remarkably improved photocatalytic H_2_ production, which was observed by photocurrent measurements and EIS studies;
(5) Therefore, the resultant MoSe_2_-WS_2_ is a capable photocatalyst for the H_2_ energy applications. Under LED light irradiation, the MoSe_2_-WS_2_ nanostructure demonstrated enhanced photocatalytic hydrogen evolution, which is approximately 3- and 10-times higher compare to bare MoSe_2_ and WS_2_. MoSe_2_-WS_2_ nanocomposite exhibits a high PHE rate of 1600 µmol g^−1^ h^−1^;
(6) The photocatalytic activity of the MoSe_2_-WS_2_ nanostructure can be explained by Z-scheme carrier transfer pathways, which favor the production of reactive species;
(7) The MoSe_2_/WS_2_ heterostructure displayed excellent electrocatalytic hydrogen evolution behavior. The demonstrated hydrogen evolution reaction performance attests to the capability of this nanohybrid to replace the high-cost and scarce Pt and will spark hybrid-based research toward the various future energy sectors. The edges of MoSe_2_ and WS_2_ present an ideal hydrogen-binding energy, which makes them promising to replace the Pt-based electrocatalysts for hydrogen generation. In addition, the MoSe_2_/WS_2_ heterostructure could be a new cost-effective electrode replacing carbon supported Pt and Pt/Ru electrodes in fuel cells.

## Figures and Tables

**Table 1:** BET analysis results of pristine WS_2_ and MoSe_2_-WS_2_ nanostructures.

Sample	BET Surface Area (m^2^ g^−1^)	Pore Volume (cm^3^ g^−1^)	Pore Size (nm)
WS_2_	106.2	0.214	10.7(2)
MoSe_2_	35.07	0.11	8.3(2)
MoSe_2_-WS_2_	132.8	0.268	10.6(5)

## Data Availability

Data is contained within the article.
